# Protective effect of nobiletin on isolated human islets survival and function against hypoxia and oxidative stress-induced apoptosis

**DOI:** 10.1038/s41598-019-48262-6

**Published:** 2019-08-12

**Authors:** Somayeh Keshtkar, Maryam Kaviani, Zahra Jabbarpour, Bita Geramizadeh, Elahe Motevaseli, Saman Nikeghbalian, Alireza Shamsaeefar, Nasrin Motazedian, Ismail H. Al-Abdullah, Mohammad Hossein Ghahremani, Negar Azarpira

**Affiliations:** 1 Department of Molecular Medicine, School of Advanced Technologies in Medicine, Tehran University of Medical Sciences, Tehran, Iran; 2 Transplant Research Center, Shiraz University of Medical Sciences, Shiraz, Iran; 3 Shiraz Organ Transplant Center, Shiraz University of Medical Sciences, Shiraz, Iran; 4 Department of Translational Research and Cellular Therapeutics, Diabetes and Metabolism Research Institute, Beckman Research Institute of City of Hope, Duarte, USA; 5 Department of Pharmacology-Toxicology, Faculty of Pharmacy, Tehran University of Medical Sciences, Tehran, Iran; 6 Shiraz Institute of Stem Cell and Regenerative Medicine, Shiraz University of Medical Sciences, Shiraz, Iran

**Keywords:** Type 1 diabetes, Drug development, Molecular medicine, Translational research, Drug development

## Abstract

Islets transplantation, as a treatment of type 1 diabetes, faces challenges, including the loss of islets in the process of isolation and pre-transplantation due to cellular stresses-induced apoptosis. Accordingly, the optimization of culture plays a decisive role in the transplantation success. In this study, we evaluated the effect of nobiletin on the cultured human islets. Isolated human islets were treated by different concentrations of nobiletin and cultured for 24 and 72 hours. Then, the islets viability, apoptosis, insulin and C-peptide secretion, and apoptosis markers were evaluated. Also, the production of reactive oxygen species (ROS), hypoxia inducible factor 1 alpha (HIF-1α), and its target genes in the islets were examined. Our findings showed that the islets were encountered with hypoxia and oxidative stress after isolation and during culture. These insults induced apoptosis and reduced viability during culture period. Moreover, the secretion of insulin and C-peptide decreased. Nobiletin treatments significantly improved the islets survival through reduction of HIF-1α and ROS production and suppression of apoptosis, along with increased islets function. Islet protective effect of nobiletin might be related to its anti-oxidant, anti-apoptotic and insulinotropic properties. Hence, in order to achieve viable and functional islets for clinical transplantation, the application of nobiletin during pre-transplantation period is useful.

## Introduction

Diabetes is a rising global health problem. It is estimated that more than 400 million people are afflicted with diabetes worldwide^1^. Type 1 diabetes involves about 5% to 10% of diabetes patients and is followed by a degenerative autoimmune reaction in β-cells of pancreatic Langerhans, leading to destruction of insulin production. Hence, type 1 diabetes patients need daily insulin injection to regulate the glycometabolic system, but few patients suffer from brittle diabetes and do not have an appropriate quality of life. On the other hand, insulin therapy does not prevent the complications of diabetes like retinopathy, nephropathy, and cardiovascular diseases especially in these group of patients^2,3^. Therefore, finding alternative approaches for treatment of type 1 diabetes patients is essential. Pancreatic islet transplantation could be an alternative and promising option for type 1 diabetes patient’s treatment. The first report of the islet transplantation in humans is related to the 1980s, which was further developed in the later years and has gained successful outcomes^4^. Nevertheless, there are several limitations in extensive application of islet transplantation.

Native islets typically possess a dense capillary network which prepares oxygen and nutrients in the islets, and β-cells consume large amounts of oxygen during insulin secretion. Accordingly, oxygen dependent β-cells make them very sensitive to hypoxic and oxidative conditions^5^. The major regulator of cellular stress responses is hypoxia inducible factor 1 alpha (HIF-1α). During hypoxia, HIF-1α activates a series of genes involved in cell adaptation pathways such as survival, angiogenesis, metabolism, and apoptosis^6^. Moreover, overproduction of reactive oxygen species (ROS), known as oxidative stress marker, produces under-hypoxic condition^7–9^. ROS and HIF-1α are also correlated together in stressful conditions, and ROS helps HIF-1α stabilization^10^.

It has been revealed that following ischemic injury of the pancreas due to disconnection from the whole body and during the isolation process, the islets encounter multiple stresses including oxidative stress and hypoxia, which continues during the pre-transplant culture period. These factors seem to be one of the main mediators in the activation of apoptosis, which can affect the islet transplantation outcome because of the low number of viable and functional islets^11–13^.

Since most transplant centers incubate the islets for 24 to 72 hours in culture media in order to control the quality of islets and preparation of recipients^14,15^, one of the critical steps in achieving a successful transplant is optimizing pre-transplant culture condition. A notable strategy to ameliorate the viable and functional islets is the use of cytoprotective agents in the culture period. Nobiletin (5, 6, 7, 8, 3′, 4′-hexamethoxyflavone) is a polymethoxylated flavonoid found in the citrus fruits and is used in traditional Chinese medicine. Nobiletin is considered by many researchers because of its widespread pharmacological properties such as anti-apoptotic, anti-inflammatory, anti-tumor, anti-oxidant, and anti-diabetes^16,17^. Moreover, several studies have represented that nobiletin has protective effects on the nervous system^18^, heart^19,20^, and kidney^21^. Findings have suggested that nobiletin protects the cells from apoptosis through activation of cyclic AMP-responsive element binding protein (CREB) and PI3/Akt pathways. These pathways promoted their target molecules including brain-derived neurotrophic factor (BDNF) and B-cell lymphoma 2 (Bcl-2), and inhibited Bcl-2-associated X protein (Bax) and caspase-3^16,21–24^. It has also been reported that nobiletin reduces the oxidative stress-induced apoptosis via decreasing ROS and increasing the anti-oxidant enzyme activities^21,25,26^.

Therfore, it is logical to consider strategy to reduce stresses-induced cell death and increase the viability and functionalty of the isolated islets to maintain them in the culture preiod. In the present study, we have studied the effect of nobiletin on human pancreatic islets.

## Results

### Nobiletin ameliorated islet survival in culture

**Figure 1:**
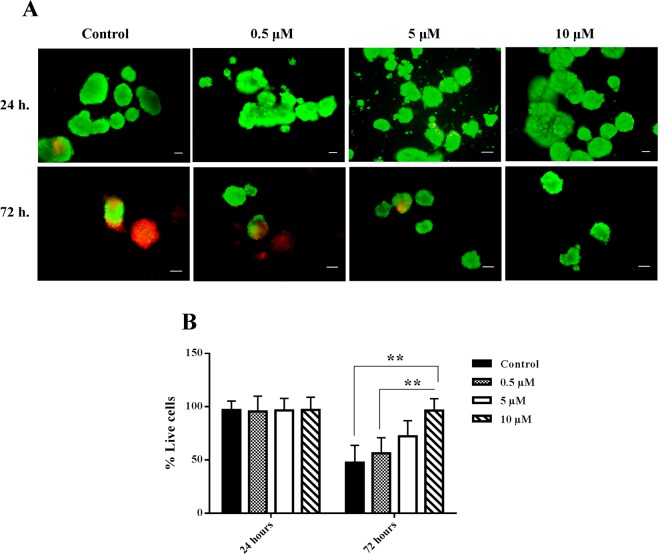
Islet viability evaluation was done by Fluorescein diacetate and propidium iodide staining in different concentrations of nobiletin. (**A**) Green cells were alive and red cells were dead. Scale bars = 100 and 200 *μ*m. (**B**) The percentage of live cells was also calculated and shown as charts. The experiment was done in triplicate from four donors and data were analyzed by Kruskal-Wallis and Post-hoc tests. The results were represented as mean ± SD. **p < 0.01.

The evaluation of islets showed that the percentage of viable islets was nearly 100% in the treatment and control groups at 24 hours, while the viability rate was reduced to fewer than 50% in the control group during 72 hours. Treatment with nobiletin increased the viable islets, which was significant in 10 µM concentration (Fig. 1). Thus, the percentage of recovery was about 50–60%.

### Nobiletin reduced islets apoptosis in culture

**Figure 2:**
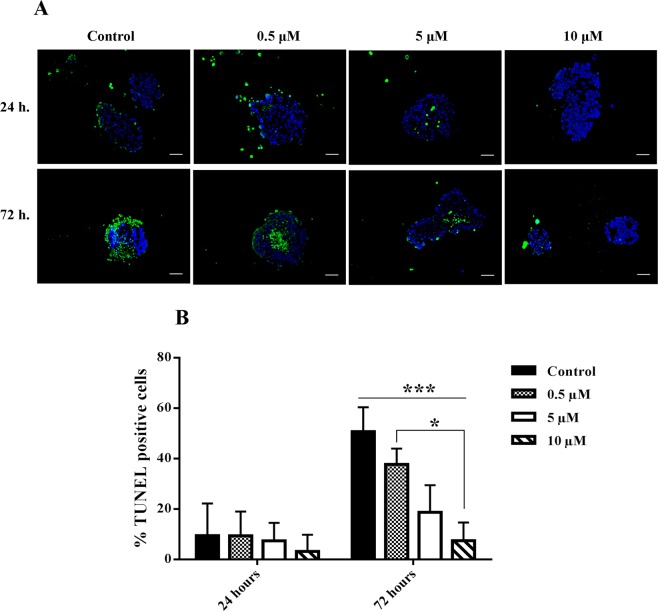
Islet apoptosis evaluation with TUNEL assay in different concentrations of nobiletin. (**A**) The TUNEL positive cells are visible as green stained nuclei. Scale bar = 100 *μ*m. (**B**) The percentage of TUNEL positive cells was also calculated and shown as graphs. The experiment was done in triplicate from four donors and data were analyzed by Kruskal-Wallis and Post-hoc tests. Results were represented as mean ± SD. *p < 0.05, and ***p < 0.001.

Apoptotic cells were detected by TUNEL assay based on DNA fragmentation within the islets. Reduction of apoptotic cells was revealed in the presence of nobiletin in different groups during 24 and 72 hours culture. However, this decrement was significant only in 10 μM nobiletin in comparison with the control group at 72 hours (Fig. 2).

### Nobiletin changed the anti-apoptotic and pro-apoptotic molecules expression in the cultured islets

**Figure 3:**
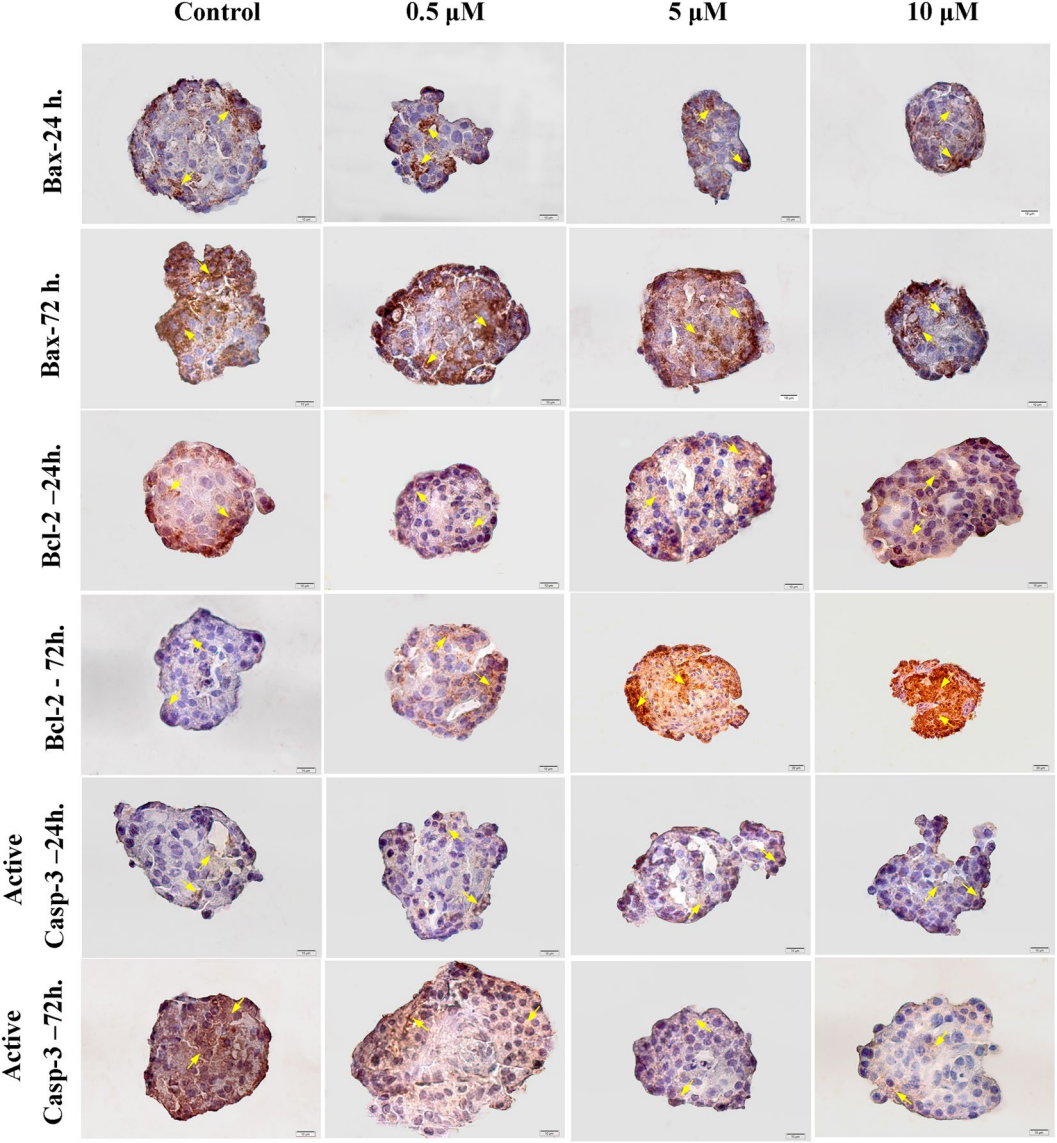
Bax, Bcl-2, and active Caspase-3 immunocytochemistry in the islets in different concentrations of nobiletin (Yellow arrows show positive protein expression). Nobiletin decreased Bax, active Caspase-3 protein level, and increased Bcl-2 protein level, especially at 72 hours. The experiment was done in triplicate from four donors. Scale bar = 10 µm and 20 µm.

**Figure 4:**
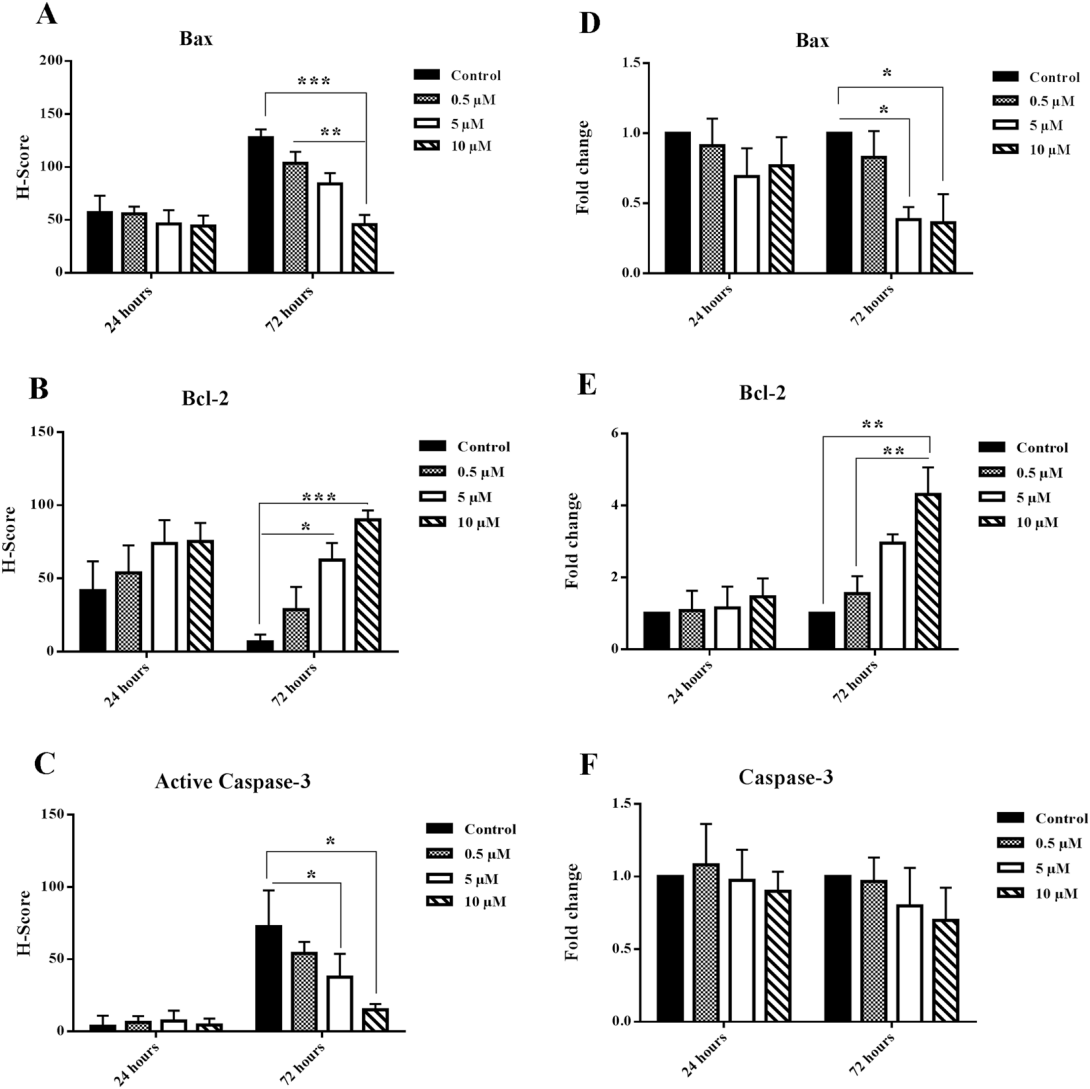
Pro-apoptotic and anti-apoptotic protein and gene expression. (**A**–**C**) Bax, Bcl-2, and active Caspase-3 protein in the islets in different concentrations of nobiletin that were calculated with H-score method, based on the percentage and intensity of the brown stained area in the islets. (**D**–**F**) Bax, Bcl-2, and Caspase-3 mRNA levels in different concentrations of nobiletin. The experiment was done in triplicate from four donors and data were analyzed by Kruskal-Wallis and Post-hoc tests. Results were represented as mean ± SD. *p < 0.05, **p < 0.01, and ***p < 0.001.

To confirm the effect of nobiletin on the islets survival, we investigated Bcl-2, Bax, and active Caspase-3 protein expression during 24 and 72 hours by immunocytochemistry and the calculated H-scores were shown as histograms (Figs 3 and 4A–C). Bcl-2 as an anti-apoptotic molecule, Bax as pro-apoptotic molecule, and active caspase-3 as an enzyme in the final stages of apoptosis were measured. Nobiletin treatment in a dose-dependent manner increased Bcl-2 protein, while it decreased Bax and active caspase-3 protein during culture periods (Fig. 3). The expression of Bax protein significantly reduced in 10 µM concentration (Fig. 4A) and the Bcl-2 level significantly increased in 5 µM and 10 µM concentrations at 72 hours treatment (Fig. 4B). Active Caspase-3 had no significant changes in 24 hours compared to the control group; however, during 72 hours culture the active Caspase-3 was increased in the control group and nobiletin treatment dose-dependently reduced the active caspase-3 level, indicating the inhibition of apoptosis. Reduction of Caspase-3 was significant in 5 µM and 10 µM nobiletin concentrations (Fig. 4C).

The gene expression evaluation of Bcl-2, Bax, and Caspase-3 indicated an up-regulated Bcl-2 mRNA and a down-regulation of Bax and caspase-3 mRNA during 24 and 72 hours culture in nobiletin treatment groups compared to the control group. The reduction of Bax was statistically significant in 5 µM and 10 µM, and enhancement of Bcl-2 was significant in 10 µM concentration (Fig. 4D,E). These results showed that nobiletin could rescue the islets from apoptosis and increase their viability.

### Nobiletin increased C-peptide and insulin secretion in the cultured islets

**Figure 5:**
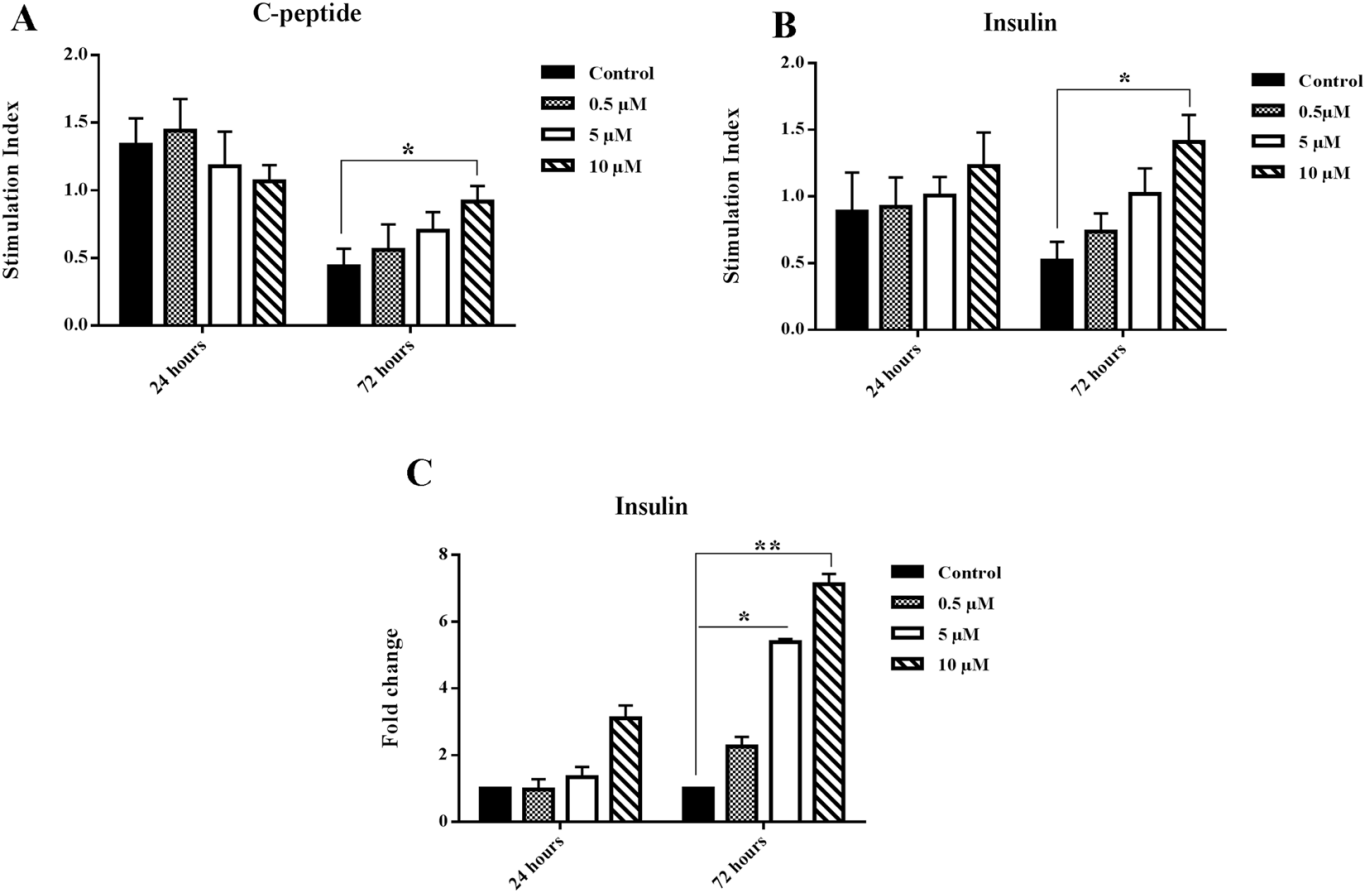
Glucose stimulation C-peptide and insulin indexes and insulin gene expression. (**A**) C-peptide and (**B**) insulin stimulation indexes and (**C**) insulin mRNA expression in different concentrations of nobiletin. The experiment was done in triplicate from four donors and data were analyzed by Kruskal-Wallis and Post-hoc tests. Results were represented as mean ± SD. *p < 0.05 and **p < 0.01.

Glucose stimulation C-peptide and insulin indexes showed no significant change in the control and nobiletin treatment groups at 24 hours. However, nobiletin treatment enhanced the C-peptide and insulin indexes compared to the control group during 72 hours incubation. This increase was significant in 10 µM concentration. The mentioned result indicated that nobiletin could improve the islet function in the culture. Furthermore, the evaluation of insulin mRNA showed that nobiletin increased the mRNA level of insulin. This increase correlated positively with up-regulation of insulin secretion. Increase of insulin mRNA level was significant in 5 µM and 10 µM of nobiletin (Fig. 5).

### Nobiletin changed HIF-1α gene and protein expression in the cultured islets

**Figure 6:**
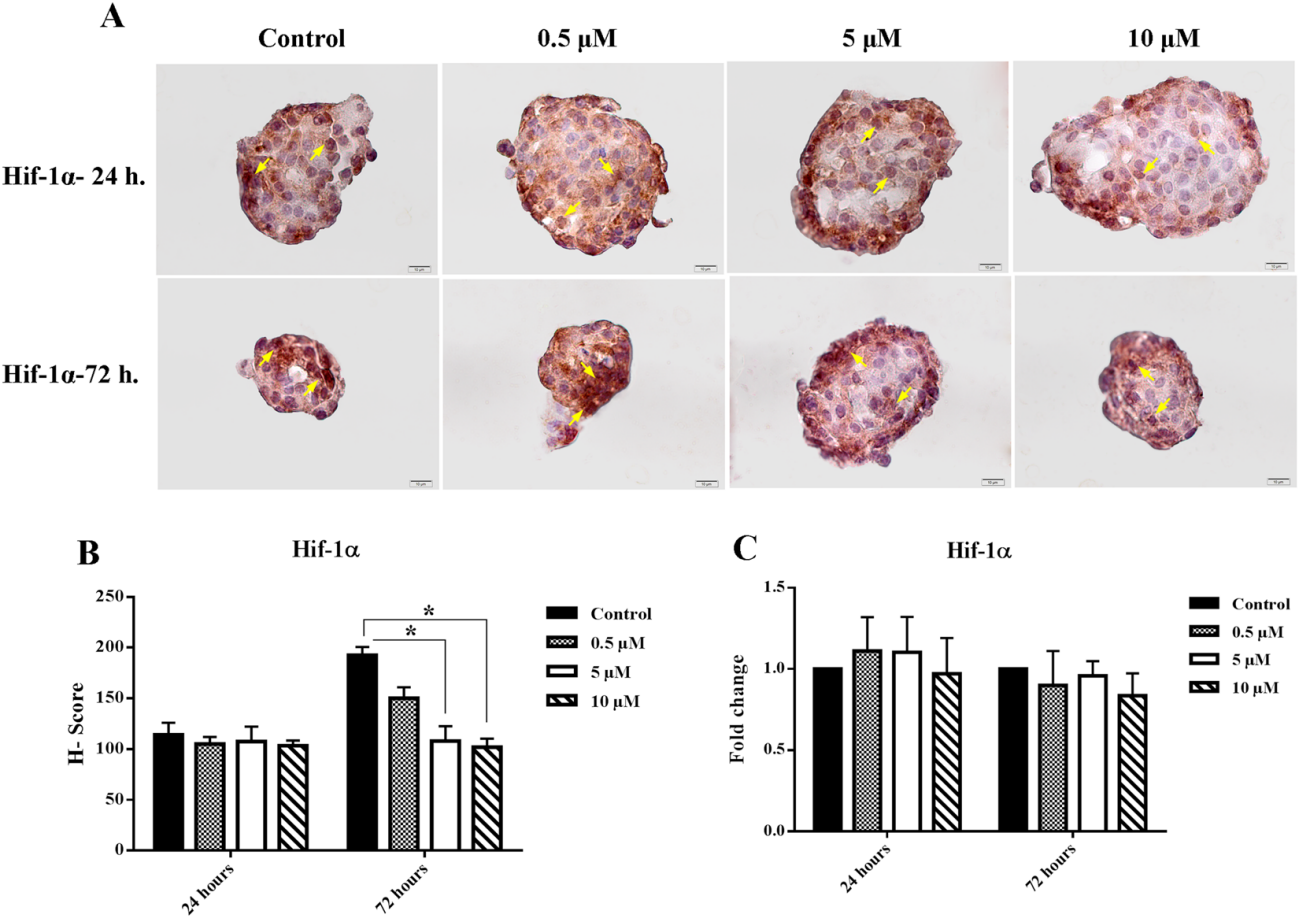
HIF-1 α protein and gene expression. (**A**) HIF-1α protein expression in the different concentrations of nobiletin. The chart shows the H-score rate based on the percentage and intensity of the brown stained area in the islets (Yellow arrows show positive protein expression). (**B**) HIF-1α gene expression in different concentrations of nobiletin. The experiment was done in triplicate from four donors and data were analyzed by Kruskal-Wallis and Post-hoc tests. Results were represented as mean ± SD. *p < 0.05. Scale bar = 10 µm.

In order to evaluate the hypoxic stress in the cultured islet, we detected the expression of HIF-1α as the main hypoxic marker by immunocytochemistry. It was found that the HIF-1α was expressed in the cultured islets. There was no difference in HIF-1α protein level between the control and nobiletin treated groups at 24 hours, whereas nobiletin reduced HIF-1α protein in the islets during 72 hours culture. Reduction of HIF-1α was significant at 5 µM and 10 µM concentrations. The gene expression evaluation of HIF-1α did not apparently change in any groups in the culture periods, suggesting that HIF-1α regulation in islets may be regulated in post-transcriptional changes at the level of protein stability under hypoxia^6,27,28^ (Fig. 6).

### Nobiletin decreased p53 protein expression in the cultured islets

**Figure 7:**
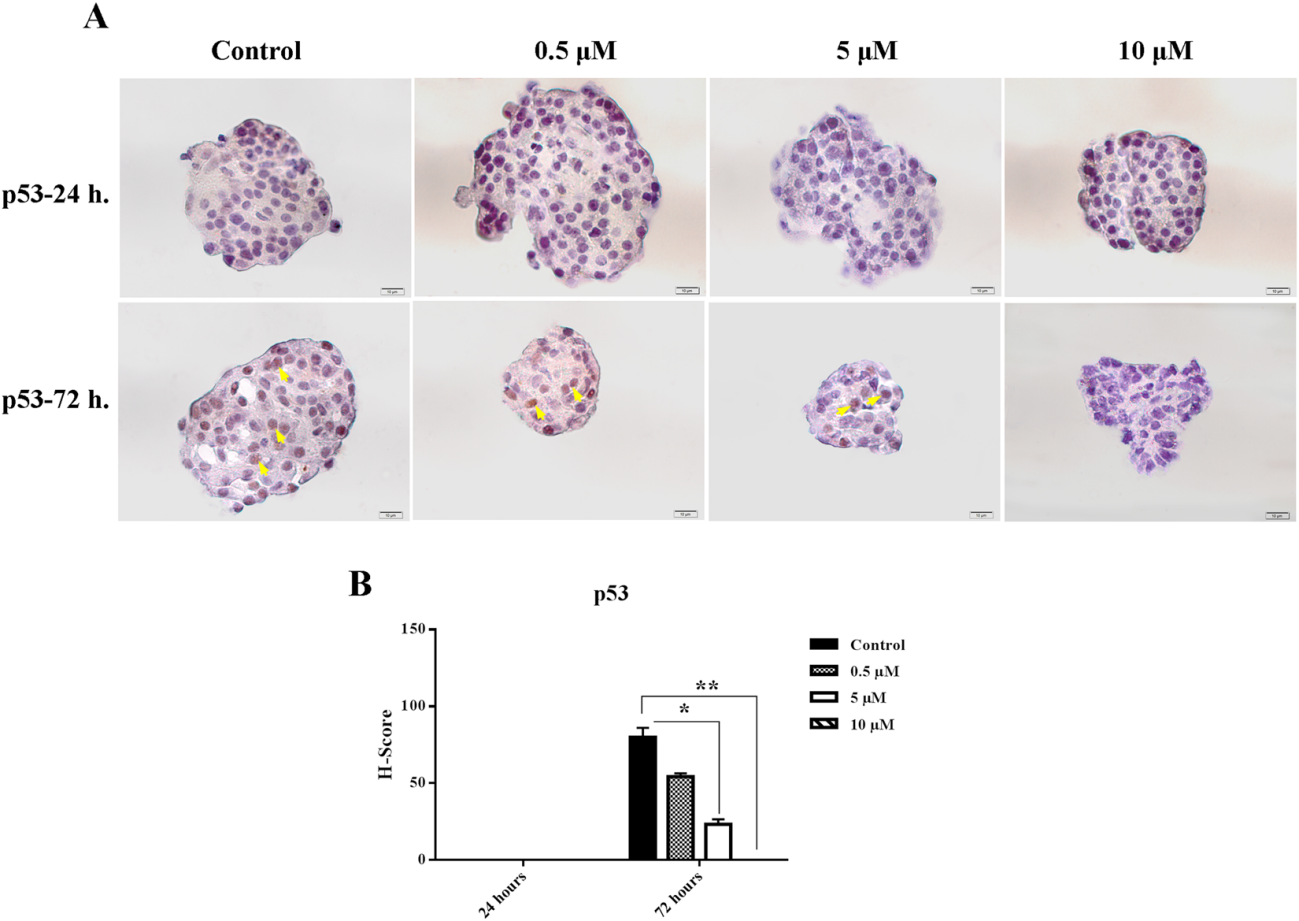
p53 protein expression (**A**) p53 protein expression by immunocytochemistry in the islets in different concentrations of nobiletin. Scale bar = 10 µm. (**B**) The chart shows the H-score rate based on the percentage and intensity of the brown stained area islets in different groups (Yellow arrows show positive protein expression). The experiment was done in triplicate from four donors and data were analyzed by Kruskal-Wallis and Post-hoc tests. Results were represented as mean ± SD. *p < 0.05 and **p < 0.01.

To demonstrate that hypoxic stress induces apoptosis, p53 protein expression was checked as a downstream molecule of HIF-1α. p53 was just expressed during 72 hours culture and nobiletin suppressed it in 5 µM and 10 µM concentrations (Fig. 7).

### Nobiletin reduced ROS production in the cultured islets

**Figure 8:**
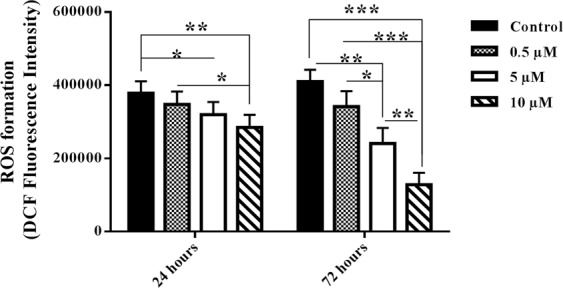
ROS measurement in the islets in different concentrations of nobiletin. The chart shows ROS formation based on DCF fluorescent intensity. The experiment was done in triplicate from four donors and data were analyzed by Kruskal-Wallis and Post-hoc tests. Result were expressed as mean ± SD. *p < 0.05, **p < 0.01, and ***p < 0.001.

Evaluation of ROS production in the islets revealed that nobiletin dose-dependently decreased it during 24 and 72 hours incubation. Diminution of ROS in the presence of nobiletin was statistically significant at 5 µM and 10 µM (Fig. 8).

### Nobiletin enhanced the VEGF secretion and gene expression in the cultured islets

**Figure 9:**
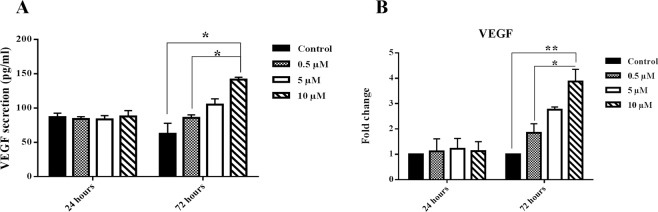
VEGF secretion and gene expression. (**A**) VEGF secretion and (**B**) VEGF mRNA expression in the islets in different concentrations of nobiletin. The experiment was done in triplicate from four donors and data were analyzed by Kruskal-Wallis and Post-hoc tests. Results were represented as mean ± SD. *p < 0.05 and **p < 0.01.

After confirming the hypoxic stress introduced into the cultured islets, we measured VEGF as a downstream target gene of HIF-1α that was expressed in mild hypoxic condition to adopt the cells with low oxygen. Our results showed that VEGF increased in mRNA and protein secretion level in the islets probably following the HIF-1α expression. However, there was a reduction in the control group at 72 hours; nobiletin treatment dose-dependently enhanced the amount of mRNA and protein secretion level of VEGF. The increase was significant in 10 µM concentration. There was, however, no apparent change between the control and nobiletin treatment groups during 24 hours incubation (Fig. 9).

## Discussion

Protection of the isolated islets and improvement of their function are necessary for successful transplantation. The widespread loss of islets during isolation and following that in the culture period is due to several insults including hypoxia and oxidative stress that can induce apoptosis. Therefore, the study of apoptotic induced mechanisms and strategies to block or diminish this process is essential for successful islet transplantation^29^.

In this study, we used nobiletin as a pharmacological approach to mitigate human pancreatic islet apoptosis and improve the viability and functionality of them in the culture. It has been reported that nobiletin exerts anti-apoptotic effects through suppression of intrinsic apoptotic pathway in acute kidney injury and neurodegenerative diseases^21,22^. Our result showed that nobiletin at 10 µM concentration significantly improved the islet survival. Furthermore, during 72 hours, nobiletin inhibited the Bax and caspase-3 and increased the Bcl-2 expression at both mRNA and protein levels. These findings indicated that the dead of islets through apoptosis reduced by nobiletin treatment.

Our observations were in agreement with other studies on nobiletin. It was shown that nobiletin improved the cisplatin-induced kidney injury through decreasing Bax and Caspase-3 and increasing Bcl-2 expression^21^. Similarly, it was reported that nobiletin protected against ischemic cerebral injury^22^ and improved isoflurane-induced cognitive impairment^23^ in rats via up-regulation of Bcl-2 expression and activation of Akt,CREB and BDNF pathways. Since the islets show some of the characteristics of neuronal phenotype due to having an endodermal lineage^30^, the suggestive protection mechanism of nobiletin may be through Akt/CREB pathway that should be investigated on the islets.

In the next step, we surveyed the effect of nobiletin on the β-cell function. It was found that nobiletin increased the insulin at both mRNA and protein secretion levels and C-peptide secretion at 72 hours. The anti-apoptotic and insulinotopic features of nobiletin on the human isolated islets are similar to glucagon-like peptide-1and prolactin^31,32^ where both these drugs reduced the number of apoptotic islets via inhibition of active Caspase-8, -9, and -3 and up-regulation of Bcl-2 in the cultured rat islets and improved the function of islets through increase in the insulin secretion^31,32^. In a recent report, nobiletin (10 µM) also inhibited β-cell apoptosis through activation of cyclic adenosine monophosphate (cAMP) pathway and downstream target protein kinase A (PKA) in INS-1D β-cell line and enhanced insulin secretion through another downstream target of cAMP called exchange proteins directly activated by cAMP (EPACs)^24^. Of note to this study, nobiletin may act via cAMP pathway in the isolated islets.

Islets are faced with hypoxia during the isolation and pre-transplant culture period because of the disconnection from the vascular network^13^. During short-term hypoxia, HIF-1α induces the expression of genes needed for metabolic adaptation including VEGF, thereby moving toward cell survival; however, in long-term hypoxia, over-aggregation of HIF-1α induces the expression of pro-apoptotic gene Bcl-2 interacting protein 3(BNIP3) or stabilizes the tumor suppressor gene p53, and moves toward apoptosis^6,7,13,33^.

Our observation showed that nobiletin (10 µM (significantly decreased the level of HIF-1α protein, scavenged ROS production, and suppressed p53 at 72 hours. The amount of VEGF mRNA and secretion also significantly increased in the presence of nobiletin. In the hypoxic response system, the VEGF expression is upregulated not only by HIF-1-mediated increase in gene transcription, but also via increased stability of VEGF mRNA, and increased VEGF protein export^29,34,35^. In our study, the HIF-1α was decreased by nobiletin at 72 hours; however, the increase in VEGF protein was remained. With this in mind, there could be other pathways involved in nobiletin induced increase in VEGF besides HIF-1α. In the current study, it was confirmed that the islets after isolation encountered hypoxia and oxidative stress that continued in the culture period. The stabilization of HIF-1α protein following hypoxia induced the expression of VEGF mRNA that could improve the islets survival during 24 hours culture because VEGF is an important growth factor for revascularization of the islets, especially after transplantation^29^. Our observations were similar to the effect of liraglutide on the isolated rat islets where this drug promoted the islet survival via enhancement of VEGF expression by activation of mammalian target of rapamycin (mTOR) pathway^36^, suggesting perhaps nobiletin was acting through this important pathway in angiogenesis.

At 72 hours culture, intracellular hypoxic in the islets led to chronic aggregation of HIF-1 α and stabilization of p53 that shifted the cells toward mitochondrial apoptosis pathway. It has been reported that the aggregation of HIF-1α protein is considered as an indicator of hypoxia that correlated with islet apoptosis^6,29,37,38^. According to our study, it seems that the duration of hypoxia and amount of HIF-1α protein affects the islet survival and death that would be confirmed with HIF-1α knockdown during different culture times.

Hypoxia also induces oxidative stress in the cells through overproduction of ROS^8,10^. Previous studies have shown that overproduction of ROS in the isolated islet can lead to β-cell death, due to weak intrinsic anti-oxidant defense system^7^. Moreover, overproduction of ROS influences cells through two pathways; one of them is the stabilization of HIF-1α and p53 and another one is disruption of the mitochondrial membrane and cytochrome C release with subsequent caspase 8, 9 and 3 activation^39^. It has been revealed that nobiletin plays an important role in apoptosis inhibition by restoring anti-oxidant activity and reducing the production of ROS in acute kidney injury and CNS ischemia^21–23^. Thus, nobiletin might reduce the islet apoptosis through its anti-oxidant properties. The anti-oxidant feature of nobiletin on the islets was similar to exendin-4 and glutathione ethyl ester where both these drugs decreased the islet apoptosis through inhibition of ROS overproduction^7,40^.

**Figure 10:**
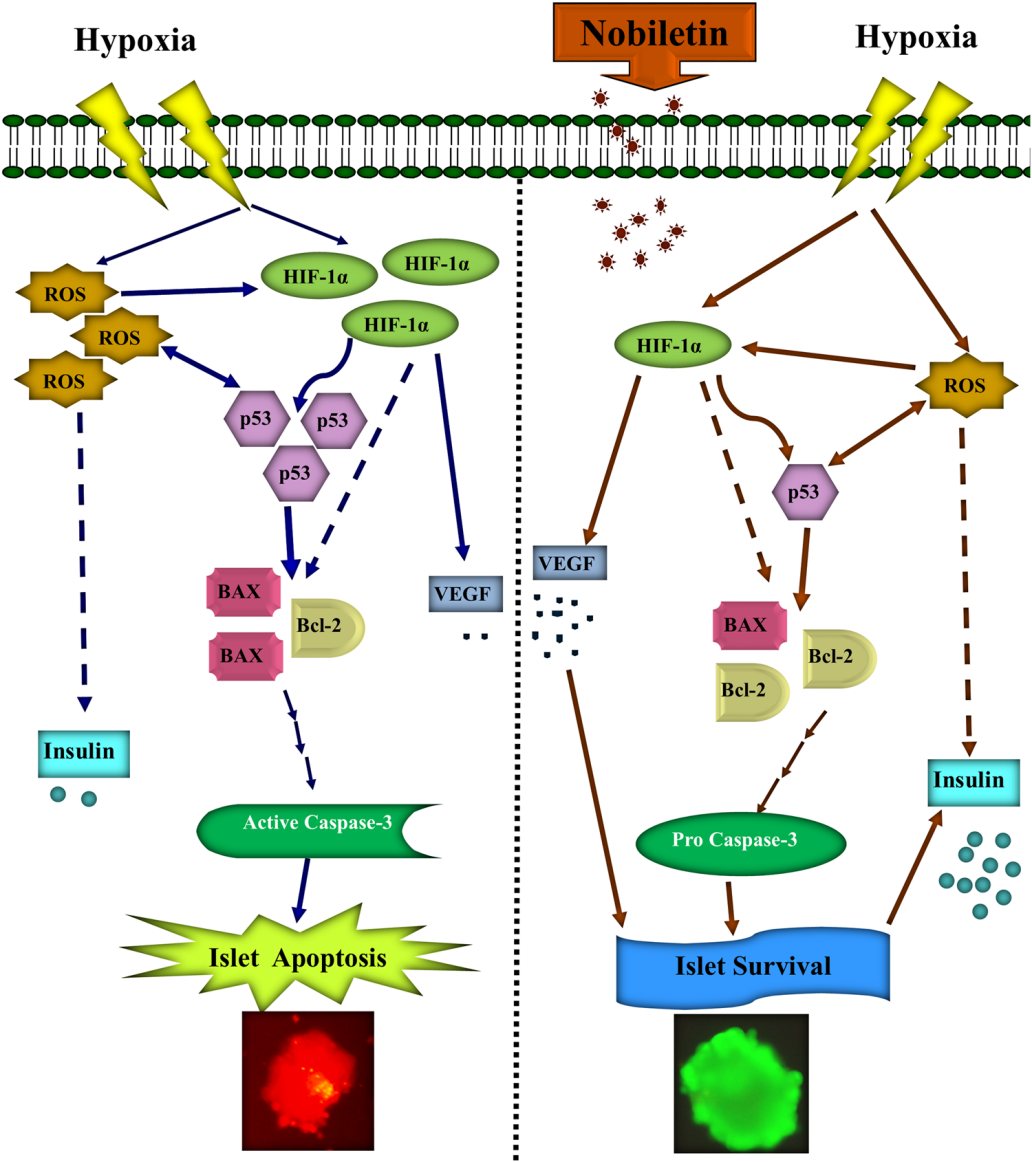
Schematic effect of nobiletin on human isolated islets in the culture. Nobiletin decreased hypoxia markers and oxidative stress induced apoptosis and improved islets survival and function.

The clinical significance of this finding is that nobiletin was able to maintain the survival of islets during 72 hours. Indeed, nobiletin helped the islets to overcome the prolonged hypoxia and oxidative stresses in the culture and in this way reduced the islets death by more than 50% and increased the insulin production and secretion. The effect of nobiletin on the isolated human islets was mediated via anti-apoptotic, insulinotropic, and anti-oxidant properties (Fig. 10). However, it would be important to investigate the capacity of the treated islets with nobiletin in diabetic animal models. Nowadays, most transplant centers keep the islets in the culture media for 24–72 hours before transplantation that allows additional time to control the quality of the islets, islet transportation to other centers, patient preparation, coordination of transplantation team, and preoperative immunosuppressive drug administration^14,15,41^. Accordingly, nobiletin is suggested as an appropriate pharmacological substance for protection of the islets in the pre-transplant culture period that will result in a large number of islets for transplantation, thereby improving the transplant outcomes.

## Conclusions

Developing protective strategies in order to overcome stress-induced apoptosis in the isolated islets would be helpful and provide higher numbers of viable and functional islets in the pre-transplant culture period, ultimately leading to successful transplantation. Nobiletin, as a cytoprotective agent, can provide an opportunity to improve the survival and function of the human pancreatic islets through its anti-apoptotic, anti-oxidant, and insulinotropic properties.

## Materials and Methods

### Human islet isolation and *in vitro* experiment

Human islets were isolated based on the semi-automated protocol of Ricordi *et al*.^42^,with a few modifications^43^. After obtaining the written informed consent for research from dead brain donors’ relatives, four human pancreases (Table 1) were taken in accordance with the institutional ethics committee (IR.TUMS.REC.1394.1306). Briefly, the pancreatic duct was distended with collagenase NB1 and neutral protease (Serva, Germany). After mechanical and enzymatic digestion in a Ricordi chamber, the islets purification was performed in a continuous Biocoll (Biochrom, Germany) gradient in a cell sorter processor called COBE 2991. The counting and purity of the islet were assessed by dithizone staining and the number of islets was expressed in the islet equivalents (IEQ)^44^. The isolated islets with more than 80% purity were used to study. They were maintained in CMRL 1066 (Gibco, UK) medium containing 1% FBS (Gibco, UK), 1% antibiotic/antimycotic (Sigma, Germany), and 6.25 µg/ml ITS (Sigma, Germany) in 5% CO2 at 37 °C for a night. Then, 700 IEQ/well islets (75 IEQ/cm^2^) were treated with nobiletin (Sigma, Germany) in three concentrations of 0.5, 5, and 10 µM^45–47^ and incubated in 5% CO_2_ at 37 °C for 24 and 72 hours. The islets without nobiletin treatment were used as the control group. The experiments were analyzed in triplicate.

**Table 1:** Characteristics of the pancreas donors.

Number	Sex	Age	Couse of death	Cold ischemic time	A history of diseases such as diabetes mellitus and cardiovascular diseases
1	Female	40	Cerebral hypoxia	2 hours	Negative
2	Male	63	Cerebrovascular accident	5 hours	Negative
3	Male	56	Head trauma	2 hours	Negative
4	Female	58	Cerebrovascular Accident	7 hours	Negative

### Islets viability evaluation

Viability of the islets was evaluated by live/dead fluorescent dyes method based on the combination of 5 mg/ml Fluorescein diacetate by live cells (Sigma, Germany) and 2 mg/ml propidium iodide by dead cells (Sigma, Germany). Images were captured under fluorescence microscope (Olympus, CKX53, Japan). The viability rate was calculated by the percentage of the fluorescein diacetate stained area to the total area.

### Islets apoptosis detection

Apoptotic islets were determined by Terminal deoxynucleotidyl transferase–mediated dUTP nick end labeling (TUNEL) assay using Click-iT^®^ Plus TUNEL Assay kit (Life technology, France) according to the manufacturer’s protocol. Nuclear counter staining was done with DAPI (Sigma, Germany). Images were captured under fluorescence microscope. The ratio between the apoptotic cells and nucleuses was used to calculate the percentage of apoptotic islets.

### Gene expression evaluation

Total RNA was extracted in each islet groups by RNA-Sol isolation kit (Alphabio, Canada), based on the manufacturer’s protocol. Then, RNA was converted to cDNA with PrimeScript TM RT Reagent Kit (Takara, Japan). Primers were designed by Primer BLAST at NCBI and sequences were: human insulin (F: 5′-CTTCTACACACCCAAGACCC-3′; R: 5′-CTGGTACAGCATTGTTCCAC-3′), human Caspase-3 (F: 5′-ACTCCACAGCACCTGGTTATT-3′; R: 5′-TCTGTTGCCACCTTTCGGTT-3′), human *Bax* (F: 5′-TTCTGACGGCAACTTCAACT-3′; R: 5′-GGAGGAAGTCCAATGTCCAG-3′), human *Bcl-2* (F: 5′-GATGGGATCGTTGCCTTATGC-3′; R: 5′-CAGTCTACTTCCTCTGTGATGTTGT-3′),human *HIF-1α* (*F*: 5′-GCAGCAACGACACAGAAACT-3′; R: 5′-TTCAGCGGTGGGTAATGGAG-3′), human vascular endothelial growth factor (*VEGF*) (F: 5′-CTTCAAGCCATCCTGTGTGC-3′; R: 5′-ATCCGCATAATCTGCATGGTG-3′) and human Glyceraldehyde 3-phosphate dehydrogenase (*GAPDH*) (F: 5′-GCTCATTTCCTGGTATGACAACG-3′; R: 5′-CTCTCTTCCTCTTGTGCTCTTG-3′), as a housekeeping gene. Relative gene expression was evaluated with real time RT-PCR by SYBR® Premix Ex TaqTM II kit (Takara, Japan) on an Applied Biosystems StepOnePlus™ System (ABI, USA). The fold changes were calculated by 2^−ΔΔCT^ for each gene. GAPDH was recruited as the internal control.

### Protein expression by immunocytochemistry assay

The islet groups were fixed in 4% paraformaldehyde and embedded in low melting agarose (Sigma, Germany). After dehydration process, the second embedding was performed in paraffin, cut in 5 μm sections, deparaffinised, and surveyed by immunocytochemistry. Immuno-labelling was done with primary antibodies specific to anti-human Bax (dilution 1:50; Abcam, France), anti-human Bcl-2 (dilution 1:250; Abcam, France), anti-human active Caspase-3 (dilution 1:50; Abcam, France), anti*-*human HIF-1α (dilution 1:50; Medaysis, USA), and anti-human p53 (dilution 1:50; Dako, Canada). All of these primary antibodies were detected by HRP- secondary antibody (dilution 1:200; Abcam, France). Finally, the samples were colored with a chromogen 3,3′- diaminobenzidine (DAB) (Dako, Canada) and counterstained with haematoxylin. The brown stained areas were scored based on the H-score method with following formula: H score = 1 × (% mild staining) + 2 × (% moderate staining) + 3 × (% strong staining)^48^.

### Glucose-stimulated insulin and C-peptide secretion

After the nobiletin treatment period, the islets were washed with PBS and RPMI 1640 without glucose (Gibco, Germany) containing 0.5% BSA, and 2.8 or 20 mM glucose was then added, and incubated in 5% CO_2_ at 37 °C for 2 hours. Both supernatants were collected and stored at −80 °C. The secreted insulin and C-peptide^49–52^ were measured with ELISA kit (Monobind, USA). In each group, the stimulation index was calculated by dividing the amount of C-peptide and the insulin released from the islets in 20 mM glucose medium into the amount of C-peptide and the insulin released from the islets in 2.8 mM glucose medium in parallel and reported as the results^40,43,53^.

### ROS measurement

Produced ROS was measured by a cell permeable and oxidation sensitive fluorescent dye 2′,7′-dichlorofluorescein diacetate (DCFH-DA) (Sigma, Germany)^54^ after the treatment period in all groups. The islets were suspended in serum-free RPMI 1640 medium and 10 μM DCFH-DA and incubated in darkness at 37 °C for 30 min. Then, the fluorescence intensity was monitored with a microplate reader (FLUOstar Omega®, BMG Labtech, Germany) with an excitation wavelength at 485 nm and an emission wavelength at 535 nm. The fluorescence intensity was normalized with total protein concentration measured by Bradford reagent at 630 nm and reported as the results^54^.

### VEGF secretion assay

After the culture period, the islet supernatants were collected and stored in at −80 °C. VEGF secretion was detected with human VEGF ELISA kit (Life technology, France) according to the manufacturer’s instruction. The results were expressed as pg/ml.

### Statistical analysis

The data were analyzed using SPSS version 24 (SPSS Inc., Chicago, IL), and the results were represented as mean ± S.D. We applied Kruskal-Wallis and Post-hoc tests to compare the groups and between the two groups, respectively. The graphs were drawn by Prism version 6 (Graph Pad Software, San Diego, California). Statistical significance was considered as P < 0.05.

### Ethics approval

Ethics approval to conduct the study was obtained from the Shiraz and Tehran University of Medical Sciences Institutional Ethics Committee (IR.TUMS.REC.1394.1306).

## Data Availability

All data generated in this study are included in the manuscript.
